# Screening of Variants in the Transcript Profile of Eutopic Endometrium from Infertile Women with Endometriosis during the Implantation Window

**DOI:** 10.1055/s-0041-1730287

**Published:** 2021-07-27

**Authors:** Michele Gomes Da Broi, Jessica Rodrigues Plaça, Wilson Araújo da Silva, Rui Alberto Ferriani, Paula Andrea Navarro

**Affiliations:** 1 Department of Gynecology and Obstetrics, Universidade de São Paulo, Ribeirão Preto, SP, Brazil

**Keywords:** endometriosis, infertility, eutopic endometrium, RNA-sequencing, mutation, endometriose, infertilidade, endométrio eutópico, sequenciamento de RNA, mutação

## Abstract

**Objective** Abnormalities in the eutopic endometrium of women with endometriosis may be related to disease-associated infertility. Although previous RNA-sequencing analysis did not show differential expression in endometrial transcripts of endometriosis patients, other molecular alterations could impact protein synthesis and endometrial receptivity. Our aim was to screen for functional mutations in the transcripts of eutopic endometria of infertile women with endometriosis and controls during the implantation window.

**Methods** Data from RNA-Sequencing of endometrial biopsies collected during the implantation window from 17 patients (6 infertile women with endometriosis, 6 infertile controls, 5 fertile controls) were analyzed for variant discovery and identification of functional mutations. A targeted study of the alterations found was performed to understand the data into disease's context.

**Results** None of the variants identified was common to other samples within the same group, and no mutation was repeated among patients with endometriosis, infertile and fertile controls. In the endometriosis group, nine predicted deleterious mutations were identified, but only one was previously associated to a clinical condition with no endometrial impact. When crossing the mutated genes with the descriptors *endometriosis* and/or *endometrium* , the gene *CMKLR1* was associated either with inflammatory response in endometriosis or with endometrial processes for pregnancy establishment.

**Conclusion** Despite no pattern of mutation having been found, we ponder the small sample size and the analysis on RNA-sequencing data. Considering the purpose of the study of screening and the importance of the *CMKLR1* gene on endometrial modulation, it could be a candidate gene for powered further studies evaluating mutations in eutopic endometria from endometriosis patients.

## Resumo

**Objetivo** Anormalidades no endométrio eutópico de mulheres com endometriose podem estar relacionadas à infertilidade associada à doença. Embora a análise prévia de sequenciamento de RNA não tenha evidenciado expressão diferencial em transcritos endometriais de pacientes com endometriose, outras alterações moleculares poderiam afetar a síntese de proteínas e a receptividade endometrial. Nosso objetivo foi rastrear mutações funcionais em transcritos de endométrios eutópicos de mulheres inférteis com endometriose e de controles durante a janela de implantação.

**Métodos** Os dados do sequenciamento de RNA de biópsias endometriais coletados durante a janela de implantação de 17 pacientes (6 mulheres inférteis com endometriose, 6 controles inférteis, 5 controles férteis) foram analisados para a descoberta de variantes e a identificação de mutações funcionais. Um estudo direcionado das alterações encontradas foi realizado para compreender os dados no contexto da doença.

**Resultados** Nenhuma das variantes identificadas foi comum a outras amostras dentro do mesmo grupo, assim como nenhuma mutação se repetiu entre pacientes com endometriose, controles inférteis e férteis. No grupo de endometriose, foram identificadas nove mutações deletérias preditas, mas apenas uma foi previamente associada a uma condição clínica sem impacto endometrial. Ao cruzar os genes mutados com os descritores *endometriose* e/ou *endométrio* , o gene *CMKLR1* foi associado a resposta inflamatória na endometriose e a processos endometriais para estabelecimento da gravidez.

**Conclusão** Apesar de nenhum padrão de mutação ter sido encontrado, ponderamos o pequeno tamanho da amostra e a análise dos dados de sequenciamento de RNA. Considerando o objetivo do estudo de triagem e a importância do gene *CMKLR1* na modulação endometrial, este poderia ser um gene candidato para estudos adicionais que avaliem mutações no endométrio eutópico de pacientes com endometriose.

## Introduction

Endometriosis, a disease characterized by implantation and growth of endometrial tissue outside the uterine cavity, 1 2 has a high prevalence, affecting between 6 and 10% of women in reproductive age. 1 It is also frequently associated with infertility, being present in between 25 and 50% of infertile women, 3 with 30 to 50% of endometriosis patients being infertile. 3 4 5 6 However, the mechanisms underlying disease-related infertility are still poorly understood.

Evidence have suggested that changes in the endometrial receptivity, due to molecular and functional disorders in the eutopic endometrium, may be related to impaired fertility in women with endometriosis. 5 7 8 9 The success of embryonic implantation depends on an adequate embryonic development, on the arrival of a competent embryo to a receptive endometrium, and on an efficient communication between the embryo and the endometrium. 10 11 12 It is known that the human endometrium becomes receptive only during the implantation window, 10 13 14 15 16 a certain period that results from the synchronized interaction of a variety of molecules (ovarian hormones, growth factors, transcription factors, cytokines, adhesion molecules), with an important role in establishing uterine receptivity. 16 17 18 19 20 21 22 Thus, molecular changes in the eutopic endometrium of these patients could impair their endometrial receptivity, contributing to the infertility observed in women with the disease.

However, a recent comprehensive and integrated evaluation of eutopic endometria of infertile women with endometriosis, infertile and fertile controls during the implantation window through a transcriptome analysis (RNA-Seq), did not identify differentially expressed transcripts among the groups. 23 Likewise, the miRNA sequencing in the eutopic endometrium of the same patients did not find changes in those post-transcriptional regulatory molecules. 23 Together, the findings suggest that the eutopic endometrium of infertile women with the disease is molecularly similar to that of fertile women. However, the absence of alterations in mRNA and miRNA expression does not exclude the possibility of other molecular changes, with consequences for protein synthesis, which could impact the endometrial receptivity of these women. Single nucleotide variants (SNVs) are changes on a DNA sequence basis and comprise both polymorphisms (single-nucleotide polymorfisms [SNPs]) and point mutations, which may result in the wrong translation of transcripts into truncated, inactive and/or altered proteins. 24 25 Since no study to date has evaluated SNVs in the eutopic endometrium of infertile women with endometriosis, we question whether the occurrence of functional mutations in the eutopic endometrium of those patients could impact the endometrial receptivity and contribute to disease-related infertility.

Total genome and/or exome sequencing are methodologies that allow the identification of point mutations in the DNA strands; however, with the disadvantage of having a high cost. 26 RNA sequencing can be a less costly alternative for the indirect study of mutations in transcripts, with the possibility of analyzing new variations that have occurred as a result of post-transcriptional changes. 27 In this sense, the use of data generated by RNA-Seq has been proposed by the literature for the indirect analysis of SNVs and mutations. 28 29 30 31 32

Thus, the objectives of the present study were to screen for functional mutations in the transcripts of eutopic endometria of infertile women with endometriosis, and of infertile and fertile controls during the implantation window, through the analysis of data previously generated by RNA-Seq, as well as to conduct a targeted study of the changes found in the context of endometriosis.

## Methods

### Study Design

A prospective case-control study was performed at the Human Reproduction Division of the Hospital das Clínicas da Faculdade de Medicina de Ribeirão Preto, Universidade de São Paulo (HCFMRP-USP). The study was approved by the Research Ethics Committee of the Hospital das Clínicas da Faculdade de Medicina de Ribeirão Preto, Universidade de São Paulo (HCFMRP-USP) (grant number 6383/2011). Patients who met the inclusion criteria and expressed their desire to participate in the study signed the informed consent form prior to inclusion.

From November 2011 to November 2014, patients previously submitted to diagnostic videolaparoscopy or tubal ligation procedures in the Hospital das Clínicas da Faculdade de Medicina de Ribeirão Preto, Universidade de São Paulo (HCFMRP-USP) were evaluated according to the eligibility criteria, and those considered eligible were interviewed. Patients who agreed to participate had an endometrial sample collected during the implantation window.

### Patients – Eligibility Criteria

We considered eligible those patients who presented regular cycles (every 24 to 38 days, 4.5 to 8 days of duration and flow up to 80 ml per cycle) 33 for at least 3 months prior to the study, aged between 18 and 45 years old, body mass index (BMI) ≤ 30 kg/m ^2^ , absence of polycystic ovary syndrome and of other etiologies of chronic anovulation, hydrosalpinx and chronic diseases such as diabetes mellitus or other endocrinopathies, cardiovascular disease, dyslipidemia, systemic lupus erythematosus and other rheumatologic diseases, HIV infection, any active infection, alcohol, drugs or smoking habit, and use of hormonal medication or of anti-inflammatory drugs during the 3 months preceding the beginning of the study were included.

In the END group, 6 patients with infertility exclusively associated to pelvic endometriosis diagnosed and classified by videolaparoscopy according to the criteria of the American Society for Reproductive Medicine 34 were included. Among them, 2 patients were diagnosed with stage I endometriosis, 1 with stage II endometriosis, 1 with stage III endometriosis and 2 with stage IV endometriosis.

In the IC group, 6 patients with infertility attributable to male and/or tubal factors who had ruled out endometriosis and other pelvic diseases by videolaparoscopy were included. The FC group was composed by 5 patients undergoing tubal ligation who were proven fertile (at least one living child) without possible associated endometrial factors.

### Sample Collection and RNA-sequencing

The patients had endometrial samples collected during the implantation window 35 (between the 20 ^th^ and 24 ^th^ days of the cycle). For data standardization, the ovulation day was considered as the 14 ^th^ day of a 28-day menstrual cycle.

Eutopic endometrial biopsies were collected during the implantation window from 17 patients (3 infertile women with endometriosis I/II, 3 infertile women with endometriosis III/IV, 6 infertile controls, and 5 fertile controls).

Total RNA was extracted with the RiboPure kit (Ambion, Life Technologies, Carlsbad, California, USA), treated with DNase (DNA KIT Free, Ambion - Life Technologies). Total RNA concentration was determined by spectrophotometry (NanoDrop 2000c; Thermo Scientific, Wilmington, DE, USA) at 260 nm, while total RNA integrity was evaluated with Agilent Technologies 2100 Bioanalyzer (Agilent, Santa Clara, CA, USA) according to the instructions of the manufacturer. Samples with RNA Integrity Number (RIN) ≥ 7.0 were considered appropriate. mRNA libraries were prepared using TruSeq RNA Sample Preparation v2 kit (Illumina, San Diego, CA, USA) according to the instructions of the manufacturer. RNA sequencing was performed using the commercial TruSeq SBS kit v5 kit (Illumina Inc.), as instructed by the manufacturer. In total, 17 libraries were distributed in 3 lanes and sequenced paired end (PE 2 × 101pb) in the HISEq. 2500 Illumina Platform, through High Output run. Data regarding the differential expression of transcripts were previously presented. 23

### Mutation Screening and Annotation

Mutation screening was performed on RNA-Seq data generated previously. 23 The mapping of the generated fragments (reads) was performed with STAR (Spliced Transcripts Alignment to a Reference), 36 and variant calling was performed using the Genome Analysis Toolkit (GATK; https://gatk.broadinstitute.org/hc/en-us/articles/360035531192?id=3891 ), following the best practices for variant discovery in RNA-Seq data, 37 filtered using the hard filtering method (-window 35 -cluster 3 -FS > 30.0 -QD (Quality By Depth.) < 2.0 -DP (Coverage) > 10.0). The annotation of SNPs and Indels was performed with the VarAFT tool ( https://varaft.eu/ ).

#### In Silico Analysis to Identify Functional Mutations

Functional mutations were selected based on quality and selection criteria (such as: depth > 10, genome region, variant function and register in the NCBI database dbSNP) and on the pathogenicity scores of the following *in silico* prediction tools: CADD (Combined Annotation Dependent Depletion); PROVEAN (Protein Variation Effect Analyzer); SIFT (Sort Intolerant From Tolerant) and Polyphen2. Only those classified as damaging, deleterious or possibly damaging in the 4 predictors were considered functional.

With the identification of possibly deleterious mutations, in order to interpret the data in the context of the disease, we performed a targeted study of the selected variants in NCBI databases such as Single Nucleotide Polymorphism Database (dbSNP) of Nucleotide Sequence Variation ( https://www.ncbi.nlm.nih.gov/snp/ ), which brings described polymorphisms, and ClinVar ( https://www.ncbi.nlm.nih.gov/clinvar/ ), which brings disease-associated mutations.

Specifically, regarding the endometriosis group, in order to target the changes found in the context of the disease, we conducted a search in PubMed crossing the genes related to each mutation with the descriptors *endometriosis* and/or *endometrium* .

### Statistical Analysis

An exploratory data analysis was performed by measurements of central position and dispersion and box-plot graphs. The Kruskal-Wallis test was used for the comparison of clinical characteristics (age, height, weight, and BMI) among the groups.

## Results

### Clinical Characteristics of the Patients

The patients from the endometriosis, infertile control and fertile control groups were similar in relation to age, weight, height and BMI ( Supplemental Table S1 (online only).

**Table 1:** Number and type of variants identified in the transcripts of eutopic endometrium of infertile women with endometriosis, women with tubal and/or male infertility factor (infertile control) and fertile women (fertile control) during the implantation window, from RNA-Seq data before and after application of filters

Group	Pacient ID	Variants		*Indel*		SNV		Total after filtering/ prediction
		Before filtering	After filtering/ prediction	Before filtering	After filtering/ prediction	Before filtering	After filtering/ prediction	
**Endometriosis**	1	72239	5	1286	0	70953	5	9
	2	16482	0	975	0	15507	0	
	3	14955	0	210	0	14745	0	
	4	84156	1	4743	0	79413	1	
	5	69363	2	1111	0	68252	2	
	6	146610	1	8595	0	138015	1	
**Fertile control**	1	79967	4	4694	0	75273	4	14
	2	66279	5	1505	0	64774	5	
	3	98901	2	5775	0	93126	2	
	4	157215	1	9525	0	147690	1	
	5	84380	2	4940	0	79440	2	
**Infertile control**	1	149952	2	9262	0	140690	2	19
	2	118616	4	7285	0	111331	4	
	3	97232	2	5600	0	91632	2	
	4	89246	1	5148	0	84098	1	
	5	88790	7	1906	0	86884	7	
	6	84869	3	4976	0	79893	3	

Abbreviation: SNV, single nucleotide variant.

### RNA sequencing

All samples that proceeded to RNA-Seq were evaluated for total RNA integrity in the 2100 BioanalyzerTM (Agilent Technologies) and were considered suitable for the technique (RIN ≥ 7). Paired-end libraries from the 17 RNA samples were sequenced: 6 women with endometriosis (3 with initial endometriosis and 3 with advanced endometriosis), 6 infertile controls and 5 fertile controls, distributed in 3 lanes, yielding ∼ 73 million reads each. Approximately 90% of the reads were mapped, with a phred-score > 30. Of the mapped reads, 1.5% were singleton, and 1% had multiple alignments, which have been removed from the analysis. The uniformity of reads mapped across all samples was considered good.

### Variant Discovery

The analyzes performed in the GATK, following the best practices recommended for discovering variants in RNA-Seq data identified 885,515 variants. The detailed data by sample and group are shown in Table 1 .

**Fig. 1:**
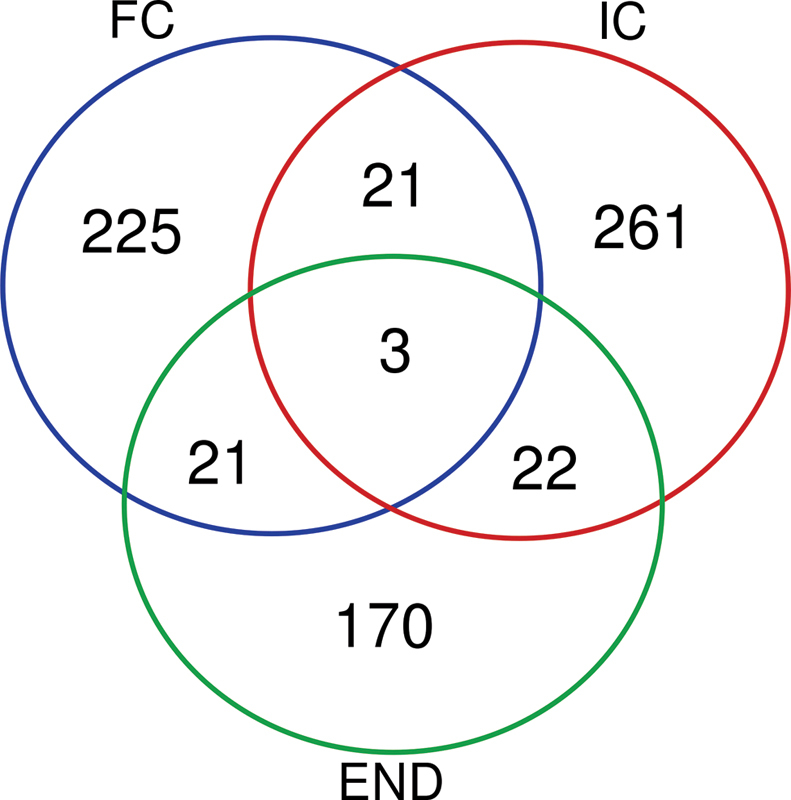
Venn diagram: number of single nucleotide variants (SNV) with depth ≥ 10, located in exonic and splicing regions, not synonymous, found in eutopic endometrial RNA-Seq data from infertile women with endometriosis (END), infertile controls (IC) and fertile controls (FC) during the implantation window.

After filtering for quality, 793 variants were identified, 225 of which were exclusive to samples from the fertile control group, 261 from the infertile control group, and 170 from the endometriosis group, in addition to the 21 common to the fertile and infertile control groups, 21 to the fertile control and endometriosis groups, 22 common to the infertile control and endometriosis groups, and 3 common to the three groups ( Fig. 1 ). According to the predictors of pathogenicity, 42 variants were selected, 14 in the fertile control group, 19 in the infertile control group, and 9 in the endometriosis group. Table 2 shows the data for the variants in each group after applying the filters. Within the endometriosis group, two samples did not present any mutation predicted as deleterious. In the other groups, all samples showed at least one mutation.

**Table 2:** Variants identified after filtering and predicting data obtained from eutopic endometrium RNA-Seq of infertile women with endometriosis, women with tubal and/or male infertility factor (infertile control), and fertile women (fertile control) during the implantation window

Group	Patient ID	Chromosome	Reference allele	Mutant allele	Genotype	Depth	SNV score	Gene	1000 g	dbSNP NCBI	CADD
**CF**	1	2	C	T	het	10	62.77	*TTN*	0.076877	rs4894028	24.0
		3	A	G	het	10	52.77	*ZNF502*	0.10603	rs56084453	17.61
		17	G	A	het	10	109.77	*EVPL*	0.0081869	rs150149800	33.0
		19	G	A	het	10	106.77	*DOCK6*	0.519569	rs12978266	22.9
	5	1	G	A	het	10	103.77	*ATAD3B*	0.00239617	rs141377718	23.5
		3	C	T	het	10	32.77	*DNAH1*	0.0299521	rs419752	34.0
		6	T	C	het	10	66.77	*GSTA3*	0.000199681	rs139422505	21.8
		8	C	A	het	10	58.77	*MAPK15*	0.095647	rs60732298	28.2
		12	A	C	het	10	71.77	*CLEC7A*	0.00858626	rs16910527	25.2
	8	1	C	T	het	10	124.77	*OXCT2*	−	rs150795467	22.6
		19	T	C	het	10	81.77	*ZNF836*	0.0129792	rs61739527	18.91
	9	1	A	C	het	10	24.78	*PLEKHN1*	−	rs181207265	20.5
	32	1	G	C	het	10	224.77	*ANKRD45*	0.00199681	rs191985325	24.7
		10	A	G	het	10	30.77	*PPP1R3C*	0.00199681	rs143318107	24.6
**CI**	2	1	C	T	het	10	127.77	*KMO*	0.000798722	rs200044625	28.8
		11	A	T	het	10	166.77	*CCDC88B*	0.000399361	rs572682028	29.4
	6	5	G	A	het	10	93.77	*PCDHB5*	0.0297524	rs17844422	18.71
		11	G	A	het	10	54.77	*SLC25A45*	0.0101837	rs34400381	26.0
		16	C	A	het	10	204.77	*MT1A*	0.470647	rs11640851	18.37
		18	G	A	het	10	69.77	*ALPK2*	0.0203674	rs79863383	24.1
	7	1	C	G	het	10	56.77	*TRAF3IP3*	0.00139776	rs147791408	22.8
		10	G	A	het	10	31.77	*CFAP58*	−	rs143080879	29.2
	17	1	G	A	het	10	67.77	*C1orf87*	−	rs772501233	26.5
	19	3	G	A	het	10	234.77	*CCDC13*	0.167732	rs17238798	24.8
			C	G	het	10	59.77	*IQCG*	0.281749	rs67877771	26.2
		5	C	T	het	10	91.77	*C5orf51*	0.00159744	rs151191974	33.0
		6	T	C	het	10	190.77	*CRYBG1*	0.0201677	rs61741114	27.0
			G	A	het	10	113.77	*LAMA4*	0.0309505	rs11757455	34.0
		11	C	T	het	10	152.77	*RIN1*	0.0183706	rs140145986	24.7
		17	G	A	het	10	94.77	*ITGAE*	0.265375	rs1716	25.0
	22	8	C	T	het	10	184.77	*MICU3*	0.000399361	rs201776772	26.8
		9	G	A	het	10	140.77	*FAM166B*	0.0333466	rs75679360	33.0
		12	G	C	het	10	49.77	*CAPRIN2*	0.0111821	rs73079976	28.0
**END**	3	4	C	T	het	10	136.77	*NSG1*	0.00139776	rs142822048	32.0
		12	G	A	het	10	111.77	*CMKLR1*	0.000199681	rs201809939	29.0
		14	G	A	hom	10	241.41	*AHNAK2*	0.538538	rs10438247	24.7
		17	A	T	het	10	108.77	*EFCAB13*	0.0892572	rs72825679	24.7
		20	T	C	het	10	97.77	*DHX35*	0.014976	rs36053162	23.0
	27	4	C	T	het	10	227.77	*SLC2A9*	0.294129	rs3733591	22.8
	28	17	G	A	het	10	44.77	*ASB16*	0.0141773	rs74491716	24.2
		19	A	T	het	10	131.77	*IZUMO4*	0.0107827	rs45506200	25.6
	31	5	C	T	het	10	224.77	*JMY*	0.0141773	rs116121324	24.5

Abbreviations: Hom, Homozygous; het, heterozygous; 1000 g, frequency described in the 1000 Genomes bank.

#### Targeted Study of Variants Found

The search of functional mutations was, then, performed in the dbSNP and ClinVar databases. The general data for each variant are presented in Table 3 . All the mutations found were classified as missense.

**Table 3:** Data from the dbSNP and ClinVar databases for the predicted pathogenic variants identified in eutopic endometrial RNA-Seq data from fertile women (fertile control; FC), women with tubal and/or male infertility factor (infertile control; IC), and infertile women with endometriosis (END) during the implantation window

Group	ID	Chr	Ref	Mut	NCBI register	Gene Symbol	Official name	Codon impact	Molecular consequence (dbSNP)	Interpretation (ClinVar)	Associated condition (ClinVar)
**CF**	1	2	C	T	rs4894028	*TTN*	titin	R (Arg) > H (His)	Missense variant	Benign / Likely benign	Dilated Cardiomyopathy, Myopathy, Hypertrophic cardiomyopathy, Limb-Girdle Muscular Dystrophy, Distal myopathy Markesbery-Griggs type
		3	A	G	rs56084453	*ZNF502*	zinc finger protein 502	Q (Gln) > R (Arg)	Missense variant	NR	−
		17	G	A	rs150149800	*EVPL*	envoplakin	R (Arg) > C (Cys)	Missense variant	NR	−
		19	G	A	rs12978266	*DOCK6*	dedicator of cytokinesis 6	P (Pro) > L (Leu)	Missense variant	Benign	Adams-Oliver syndrome 2
	2	1	G	A	rs141377718	*ATAD3B*	ATPase family AAA domain containing 3B	V (Val) > M (Met)	Missense variant	NR	−
		3	C	T	rs419752	*DNAH1*	dynein axonemal heavy chain 1	R (Arg) > C (Cys)	Missense variant	Benign	• Ciliary dyskinesia, Spermatogenic failure
		6	T	C	rs139422505	*GSTA3*	glutathione S-transferase α 3	N (Asn) > S (Ser)	Missense variant	NR	−
		8	C	A	rs60732298	*MAPK15*	Mitogen-activated protein kinase 15	T (Thr) > K (Lys)	Missense variant	NR	−
		12	A	C	rs16910527	*CLEC7A*	C-type lectin domain containing 7A	I (Ile) > S (Ser)	Missense variant	NR	−
	3	1	C	T	rs150795467	*OXCT2*	3-oxoacid CoA-transferase 2	D (Asp) > N (Asn)	Missense variant	NR	−
		19	T	C	rs61739527	*ZNF836*	zinc finger protein 836	E (Glu) > G (Gly)	Missense variant	NR	−
	4	1	A	C	rs181207265	*PLEKHN1*	pleckstrin homology domain containing N1	T (Thr) > P (Pro)	Missense variant	NR	−
	5	1	G	C	rs191985325	*ANKRD45*	ankyrin repeat domain 45	L (Leu) > V (Val)	Missense variant	NR	−
		10	A	G	rs143318107	*PPP1R3C*	protein phosphatase 1 regulatory subunit 3C	F (Phe) > S (Ser)	Missense variant	NR	−
**CI**	1	1	C	T	rs200044625	*KMO*	kynurenine 3-monooxygenase	T (Thr) > I (Ile)	Missense variant	NR	−
		11	A	T	rs572682028	*CCDC88B*	coiled-coil domain containing 88B	E (Glu) > V (Val)	Missense variant	NR	−
	2	5	G	A	rs17844422	*PCDHB5*	protocadherin β 5	S (Ser) > N (Asn)	Missense variant	NR	−
		11	G	A	rs34400381	*SLC25A45*	solute carrier family 25 member 45	R (Arg) > C (Cys)	Missense variant	NR	−
		16	C	A	rs11640851	*MT1A*	metallothionein 1A	T (Thr) > N (Asn)	Missense variant	NR	−
		18	G	A	rs79863383	*ALPK2*	α kinase 2	T (Thr) > I (Ile)	Missense variant	NR	−
	3	1	C	G	rs147791408	*TRAF3IP3*	TRAF3 interacting protein 3	D (Asp) > E (Glu)	Missense variant	NR	−
		10	G	A	rs143080879	*CFAP58*	cilia and flagella associated protein 58	R (Arg) > H (His)	Missense variant	NR	−
	4	1	G	A	rs772501233	*C1orf87*	chromosome 1 open reading frame 87	A (Ala) > V (Val)	Missense variant	NR	−
	5	3	G	A	rs17238798	*CCDC13*	coiled-coil domain containing 13	R (Arg) > W (Trp)	Missense variant	NR	−
		3	C	G	rs67877771	*IQCG*	IQ motif containing G	D (Asp) > H (His)	Missense variant	NR	−
		5	C	T	rs151191974	*C5orf51*	chromosome 5 open reading frame 51	P (Pro) > L (Leu)	Missense variant	NR	−
		6	T	C	rs61741114	*CRYBG1*	crystallin β-gamma domain containing 1	L (Leu) > P (Pro)	Missense variant	NR	−
		6	G	A	rs11757455	*LAMA4*	laminin subunit α 4	R (Arg) > W (Trp)	Missense variant	Benign	−
		11	C	T	rs140145986	*RIN1*	Ras and Rab interactor 1	A (Ala) > T (Thr)	Missense variant	NR	−
		17	G	A	rs1716	*ITGAE*	integrin subunit α E	R (Arg) > W (Trp)	Missense variant	NR	−
**END**	1	4	C	T	rs142822048	*NSG1*	neuronal vesicle trafficking associated 1	P (Pro) > S (Ser)	Missense variant	NR	−
		12	G	A	rs201809939	*CMKLR1*	chemerin chemokine-like receptor 1	R (Arg) > C (Cys)	Missense variant	NR	−
		14	G	A	rs10438247	*AHNAK2*	AHNAK nucleoprotein 2	P (Pro) > L (Leu)	Missense variant	NR	−
		17	A	T	rs72825679	*EFCAB13*	EF-hand calcium-binding domain-containing protein 13	D (Asp) > V (Val)	Missense variant	NR	−
		20	T	C	rs36053162	*DHX35*	DEAH-box helicase 35	I (Ile) > T (Thr)	Missense variant	NR	−
	4	4	C	T	rs3733591	*SLC2A9*	solute carrier family 2 member 9	R (Arg) > H (His)	Missense variant	Benign	Familial renal hypouricemia
	5	17	G	A	rs74491716	*ASB16*	ankyrin repeat and SOCS box containing 16	A (Ala) > T (Thr)	Missense variant	NR	−
		19	A	T	rs45506200	*IZUMO4*	IZUMO family member 4	Y (Tyr) > F (Phe)	Missense variant	NR	−
	6	5	C	T	rs116121324	*JMY*	junction mediating and regulatory protein, p53 cofactor	P (Pro) > L (Leu)	Missense variant	NR	−

Abbreviations: Chr, chromosome; ID, patient identification; Mut, mutated allele; NR, not reported; Ref, reference allele.

According to the findings ( Table 3 ), in the fertile control group, two patients had mutations corresponding to clinical conditions. Among them, patient 1 presented two mutations with associated pathological conditions, being one related to cardiomyopathy and the other to Adams-Oliver syndrome 2, both with benign significance. Patient 2 presented one mutation related to spermatogenic failure and ciliary dyskinesia, also with benign significance. The infertile control group did not have any mutations with an associated clinical condition. In the endometriosis group, only patient 4 presented a mutation associated to a clinical condition (familial renal hypouricemia), with a benign significance.

Specifically, regarding the endometriosis group, when we performed a search in the PubMed database, by crossing the mutated genes identified with the descriptors *endometriosis* and/or *endometrium* , only the *CMKLR1* gene was associated with those descriptors. Accordingly, the protein encoded by *CMKLR1* is increased in the peritoneal fluid of women with endometriosis when compared with controls. In addition, its mRNA protein and receptor appear to be increased in ovarian endometrioma compared with the eutopic endometrium of control women.

## Discussion

Endometriosis is a disease related to infertility whose underlying mechanisms that impair the fertility of women are still under investigation. 1 An endometrial factor has been considered, since molecular and functional alterations of the eutopic endometrium could affect embryo implantation. 3 5 7 8 9 Despite a recent study that evidenced no differential expression in the mRNA and miRNA profile in the endometrium of those patients, 23 other molecular aberrations could impair protein synthesis and, consequently, endometrial receptivity. However, there is no study to date that evaluated eutopic endometrial mutations in endometriosis patients during the implantation window, which could bring important information regarding functional alterations in their endometrium. Because RNA-Seq data may be useful to identify variants in the transcriptome, 26 27 28 29 30 31 32 the aim of the present study was to screen for functional mutations in the transcripts (mRNA) of eutopic endometria of infertile women with endometriosis and of controls during the implantation window, through the analysis of data previously generated by RNA-Seq. 38

According to the findings, none of the variants found were common to other samples within the same group, suggesting no pattern of mutations in those patients. Also, no variant was repeated among women with endometriosis, infertile controls, and fertile controls. Interestingly, the endometriosis group had the lower number of variants, followed by the fertile control group, with the infertile control group having the highest number of mutations. However, it is important to highlight the small sample size of the groups, which may represent a bias and precludes groups comparison. Powered studies are necessary to confirm those results.

All the filtered mutations were classified as missense, which means that the substitution of a single base pair alters the genetic code and produces an aminoacid which is different from the usual, which is able to affect the protein function. 39 It is known that the phenotypic effects of a mutation can be more severe the greater the difference in the chemical nature of the side chains of the aminoacid residues, and that they also depend on the role that this residue plays in the structure and function of the protein. 39 Nevertheless, in the endometriosis group, only one patient presented a mutation associated with a clinical condition (familial renal hypouricemia). Renal hypouricemia is characterized by impaired reabsorption of uric acid in the apical membrane of proximal renal tubule cells caused by dysfunction of renal urate reabsorption transporters. 40 Patients are usually asymptomatic, but, in some cases, they may present exercise-induced acute renal failure and nephrolithiasis. 41 42 However, the disease has no relation with the endometrium or with infertility.

Regarding the endometriosis group, there are evidence relating one of the mutated genes ( *CMKLR1* ) with endometriosis and/or the endometrium. The *CMKLR1* gene encodes a protein called chemerin, which is an adipokine expressed in several human organs. 43 44 45 This protein has been associated with several systemic and focal inflammatory processes. 43 44 45 46 47 It modulates chemotaxis and activates inflammatory macrophages and cytokines. 48 The *CMKLR1* gene is also associated with important endometrial events for pregnancy, such as accumulation of deciduous natural killer (NK) cells and vascular remodeling. In this sense, chemerin levels seems to be higher in stromal endometrial cells of pregnant women compared with nonpregnant or menopausal fertile women, being regulated positively during decidualization. 49

Interestingly, chemerin plays a role in pelvic inflammation related to endometriosis, and its concentration is increased in the peritoneal fluid of women with the disease when compared with controls. In addition, its mRNA, protein and receptor appear to be increased in ovarian endometrioma compared with the eutopic endometrium of control women. 38 However, there is no data about the expression of *CMKLR1* in the eutopic endometrium of women with endometriosis comparing them to fertile controls. In this sense, given its role in the inflammatory process, chemerin could have a role in the impairment of fertility of those patients. The endometrial *CMKLR1* gene mutation could be involved in reduced chemotaxis, less activation of macrophages and decreased release of inflammatory cytokines. Considering that the inflammatory process is important for endometrial receptivity and embryo implantation 50 51 52 and that chemerin plays a direct role in the establishment of pregnancy, 49 it is questioned whether the mutation of the *CMKLR1* gene could be related to the impairment of those important events in women with endometriosis, being able to participate in the etiopathogenesis of disease-related infertility. However, this should be clarified in future studies with appropriate methodologies.

The present study has limitations, such as the small sample size, which does not allow us to state whether there are differential mutations among women with endometriosis compared with fertile and infertile controls, nor the identification of a pattern of mutations in the endometriosis group. Moreover, the search for variants was performed on RNA-Seq data, which may add bias by evaluating only expressed transcripts. It is unknown whether other mutations, in regulatory regions, for example, may characterize those patients and impact the phenotype.

In summary, no pattern of functional mutations was identified in the transcripts of the eutopic endometria from infertile women with endometriosis during the implantation window. However, it is necessary to consider the small sample size and that the analyses were performed on RNA-Seq data. Interestingly, one of the mutations found in one endometriosis patient was related to a gene ( *CMKLR1* ) already associated with endometriosis, endometrial function, and initial gestational development.

## Conclusion

Considering the aim of the present study of screening analysis and the importance of the *CMKLR1* gene in endometrial modulation, *CMKLR1* could be suggested as a candidate gene for further studies evaluating mutations in the eutopic endometrium from endometriosis patients. Thus, according to the present findings, future studies with appropriate casuistry, which investigate the *CMKLR1* mutation in DNA samples (and not in transcripts) and evaluate the respective protein (chemerin) in the eutopic endometria of infertile women with endometriosis may clarify this issue and contribute to the understanding of endometriosis-related infertility.

## References

[1] Burney R O Giudice L C Pathogenesis and pathophysiology of endometriosis Fertil Steril 2012 98 03 511 519 10.1016/j.fertnstert.2012.06.029 22819144 PMC3836682

[2] Gupta S Agarwal A Krajcir N Alvarez J G Role of oxidative stress in endometriosis Reprod Biomed Online 2006 13 01 126 134 10.1016/s1472-6483(10)62026-3 16820124

[3] Practice Committee of the American Society for Reproductive Medicine Endometriosis and infertility: a committee opinion Fertil Steril 2012 98 03 591 598 10.1016/j.fertnstert.2012.05.031 22704630

[4] Garrido N Navarro J Remohí J Simón C Pellicer A Follicular hormonal environment and embryo quality in women with endometriosis Hum Reprod Update 2000 6 01 67 74 10.1093/humupd/6.1.67 10711831

[5] Giudice L C Kao L C Endometriosis Lancet 2004 364(9447):1789 1799 10.1016/S0140-6736(04)17403-5 15541453

[6] Gupta S Goldberg J M Aziz N Goldberg E Krajcir N Agarwal A Pathogenic mechanisms in endometriosis-associated infertility Fertil Steril 2008 90 02 247 257 10.1016/j.fertnstert.2008.02.093 18672121

[7] Practice Committee of the American Society for Reproductive Medicine Endometriosis and infertility Fertil Steril 2006 86(05, Suppl 1):S156 S160 10.1016/j.fertnstert.2006.08.014 17055813

[8] Wei Q St Clair J B Fu T Stratton P Nieman L K Reduced expression of biomarkers associated with the implantation window in women with endometriosis Fertil Steril 2009 91 05 1686 1691 10.1016/j.fertnstert.2008.02.121 18672236 PMC2697117

[9] Bulletti C Coccia M E Battistoni S Borini A Endometriosis and infertility J Assist Reprod Genet 2010 27 08 441 447 10.1007/s10815-010-9436-1 20574791 PMC2941592

[10] Giudice L C Telles T L Lobo S Kao L The molecular basis for implantation failure in endometriosis: on the road to discovery Ann N Y Acad Sci 2002 955 252 264, discussion 293–295, 396–406 10.1111/j.1749-6632.2002.tb02786.x 11949953

[11] Minici F Tiberi F Tropea A Orlando M Gangale M F Romani F R Endometriosis and human infertility: a new investigation into the role of eutopic endometrium Hum Reprod 2008 23 03 530 537 10.1093/humrep/dem399 18096563

[12] Singh M Chaudhry P Asselin E Bridging endometrial receptivity and implantation: network of hormones, cytokines, and growth factors J Endocrinol 2011 210 01 5 14 10.1530/JOE-10-0461 21372150

[13] Kresowik J D Devor E J Van Voorhis B J Leslie K K MicroRNA-31 is significantly elevated in both human endometrium and serum during the window of implantation: a potential biomarker for optimum receptivity Biol Reprod 2014 91 01 17 10.1095/biolreprod.113.116590 24855107 PMC6322437

[14] Achache H Revel A Endometrial receptivity markers, the journey to successful embryo implantation Hum Reprod Update 2006 12 06 731 746 10.1093/humupd/dml004 16982667

[15] Bourgain C Devroey P Histologic and functional aspects of the endometrium in the implantatory phase Gynecol Obstet Invest 2007 64 03 131 133 10.1159/000101735 17934307

[16] Wilcox A J Baird D D Weinberg C R Time of implantation of the conceptus and loss of pregnancy N Engl J Med 1999 340 23 1796 1799 10.1056/NEJM199906103402304 10362823

[17] Altmäe S Esteban F J Stavreus-Evers A Simón C S Giudice L Lessey B A Guidelines for the design, analysis and interpretation of ‘omics’ data: focus on human endometrium Hum Reprod Update 2014 20 01 12 28 10.1093/humupd/dmt048 24082038 PMC3845681

[18] Díaz-Gimeno P Ruíz-Alonso M Blesa D Simón C Transcriptomics of the human endometrium Int J Dev Biol 2014 58(2-4):127 137 10.1387/ijdb.130340pd 25023678

[19] Hu S Yao G Wang Y Xu H Ji X He Y Transcriptomic changes during the pre-receptive to receptive transition in human endometrium detected by RNA-Seq J Clin Endocrinol Metab 2014 99 12 E2744 E2753 10.1210/jc.2014-2155 25243572

[20] Paulson R J Hormonal induction of endometrial receptivity Fertil Steril 2011 96 03 530 535 10.1016/j.fertnstert.2011.07.1097 21880274

[21] von Grothusen C Lalitkumar S Boggavarapu N R Gemzell-Danielsson K Lalitkumar P G Recent advances in understanding endometrial receptivity: molecular basis and clinical applications Am J Reprod Immunol 2014 72 02 148 157 10.1111/aji.12226 24635108

[22] Aghajanova L Hamilton A E Giudice L C Uterine receptivity to human embryonic implantation: histology, biomarkers, and transcriptomics Semin Cell Dev Biol 2008 19 02 204 211 10.1016/j.semcdb.2007.10.008 18035563 PMC2829661

[23] Da Broi M G Meola J Plaça J R Peronni K C Rocha C V Silva W A Is the profile of transcripts altered in the eutopic endometrium of infertile women with endometriosis during the implantation window? Hum Reprod 2019 34 12 2381 2390 10.1093/humrep/dez225 31796963

[24] Katsonis P Koire A Wilson S J Hsu T-K Lua R C Wilkins A D Single nucleotide variations: biological impact and theoretical interpretation Protein Sci 2014 23 12 1650 1666 10.1002/pro.2552 25234433 PMC4253807

[25] Mueller S C Backes C Kalinina O V Meder B Stöckel D Lenhof H-S BALL-SNP: combining genetic and structural information to identify candidate non-synonymous single nucleotide polymorphisms Genome Med 2015 7 01 65 10.1186/s13073-015-0190-y 26191084 PMC4506604

[26] Center for HIV/AIDS Vaccine Immunology (CHAVI) Cirulli E T Singh A Shianna K V Ge D Smith J P Maia J M Screening the human exome: a comparison of whole genome and whole transcriptome sequencing Genome Biol 2010 11 05 R57 10.1186/gb-2010-11-5-r57 20598109 PMC2898068

[27] Han L Vickers K C Samuels D C Guo Y Alternative applications for distinct RNA sequencing strategies Brief Bioinform 2015 16 04 629 639 10.1093/bib/bbu032 25246237 PMC4542857

[28] Sheng Q Zhao S Li C I Shyr Y Guo Y Practicability of detecting somatic point mutation from RNA high throughput sequencing data Genomics 2016 107 05 163 169 10.1016/j.ygeno.2016.03.006 27046520 PMC5663213

[29] Quinn E M Cormican P Kenny E M Hill M Anney R Gill M Development of strategies for SNP detection in RNA-seq data: application to lymphoblastoid cell lines and evaluation using 1000 Genomes data PLoS One 2013 8 03 e58815 10.1371/journal.pone.0058815 23555596 PMC3608647

[30] Chepelev I Wei G Tang Q Zhao K Detection of single nucleotide variations in expressed exons of the human genome using RNA-Seq Nucleic Acids Res 2009 37 16 e106 10.1093/nar/gkp507 19528076 PMC2760790

[31] Cánovas A Rincon G Islas-Trejo A Wickramasinghe S Medrano J F SNP discovery in the bovine milk transcriptome using RNA-Seq technology Mamm Genome 2010 21(11-12):592 598 10.1007/s00335-010-9297-z 21057797 PMC3002166

[32] Peng Z Cheng Y Tan B C Tian Z Zhu Y Zhang W Comprehensive analysis of RNA-Seq data reveals extensive RNA editing in a human transcriptome Nat Biotechnol 2012 30 03 253 260 10.1038/nbt.2122 22327324

[33] Writing Group for this Menstrual Agreement Process Fraser I S Critchley H O Munro M G Broder M A process designed to lead to international agreement on terminologies and definitions used to describe abnormalities of menstrual bleeding Fertil Steril 2007 87 03 466 476 10.1016/j.fertnstert.2007.01.023 17362717

[34] Revised American Society for Reproductive Medicine classification of endometriosis: 1996 Fertil Steril 1997 67 05 817 821 10.1016/s0015-0282(97)81391-x 9130884

[35] Noyes R W Hertig A T Rock J Dating the endometrial biopsy Am J Obstet Gynecol 1975 122 02 262 263 10.1016/s0002-9378(16)33500-1 1155504

[36] Dobin A Davis C A Schlesinger F Drenkow J Zaleski C Jha S STAR: ultrafast universal RNA-seq aligner Bioinformatics 2013 29 01 15 21 10.1093/bioinformatics/bts635 23104886 PMC3530905

[37] Van der Auwera G A Carneiro M O Hartl C Poplin R Del Angel G Levy-Moonshine A From FastQ data to high confidence variant calls: the Genome Analysis Toolkit best practices pipeline Curr Protoc Bioinformatics 2013 43(1110):1 33 10.1002/0471250953.bi1110s43 PMC4243306 25431634

[38] Jin C H Yi K W Ha Y R Shin J-H Park H T Kim T Chemerin expression in the peritoneal fluid, serum, and ovarian endometrioma of women with endometriosis Am J Reprod Immunol 2015 74 04 379 386 10.1111/aji.12405 26059828

[39] Stefl S Nishi H Petukh M Panchenko A R Alexov E Molecular mechanisms of disease-causing missense mutations J Mol Biol 2013 425 21 3919 3936 10.1016/j.jmb.2013.07.014 23871686 PMC3796015

[40] Nakayama A Matsuo H Ohtahara A Ogino K Hakoda M Hamada T Clinical practice guideline for renal hypouricemia (1st edition) Hum Cell 2019 32 02 83 87 30783949 10.1007/s13577-019-00239-3 PMC6437292

[41] Dinour D Gray N K Campbell S Shu X Sawyer L Richardson W Homozygous SLC2A9 mutations cause severe renal hypouricemia J Am Soc Nephrol 2010 21 01 64 72 10.1681/ASN.2009040406 19926891 PMC2799278

[42] Windpessl M Ritelli M Wallner M Colombi M A novel homozygous SLC2A9 mutation associated with renal-induced hypouricemia Am J Nephrol 2016 43 04 245 250 10.1159/000445845 27116386

[43] Bozaoglu K Bolton K McMillan J Zimmet P Jowett J Collier G Chemerin is a novel adipokine associated with obesity and metabolic syndrome Endocrinology 2007 148 10 4687 4694 10.1210/en.2007-0175 17640997

[44] Bozaoglu K Segal D Shields K A Cummings N Curran J E Comuzzie A G Chemerin is associated with metabolic syndrome phenotypes in a Mexican-American population J Clin Endocrinol Metab 2009 94 08 3085 3088 10.1210/jc.2008-1833 19470637 PMC2730868

[45] Bozaoglu K Curran J E Stocker C J Zaibi M S Segal D Konstantopoulos N Chemerin, a novel adipokine in the regulation of angiogenesis J Clin Endocrinol Metab 2010 95 05 2476 2485 10.1210/jc.2010-0042 20237162 PMC2869547

[46] Bondue B Wittamer V Parmentier M Chemerin and its receptors in leukocyte trafficking, inflammation and metabolism Cytokine Growth Factor Rev 2011 22(5-6):331 338 10.1016/j.cytogfr.2011.11.004 22119008

[47] Roh S G Song S H Choi K C Katoh K Wittamer V Parmentier M Chemerin--a new adipokine that modulates adipogenesis via its own receptor Biochem Biophys Res Commun 2007 362 04 1013 1018 10.1016/j.bbrc.2007.08.104 17767914

[48] Weigert J Neumeier M Wanninger J Filarski M Bauer S Wiest R Systemic chemerin is related to inflammation rather than obesity in type 2 diabetes Clin Endocrinol (Oxf) 2010 72 03 342 348 10.1111/j.1365-2265.2009.03664.x 19558533

[49] Carlino C Trotta E Stabile H Morrone S Bulla R Soriani A Chemerin regulates NK cell accumulation and endothelial cell morphogenesis in the decidua during early pregnancy J Clin Endocrinol Metab 2012 97 10 3603 3612 10.1210/jc.2012-1102 22791765 PMC3462933

[50] Maybin J A Critchley H O Jabbour H N Inflammatory pathways in endometrial disorders Mol Cell Endocrinol 2011 335 01 42 51 10.1016/j.mce.2010.08.006 20723578

[51] King A E Critchley H O Oestrogen and progesterone regulation of inflammatory processes in the human endometrium J Steroid Biochem Mol Biol 2010 120(2-3):116 126 10.1016/j.jsbmb.2010.01.003 20067835

[52] Roberts M Luo X Chegini N Differential regulation of interleukins IL-13 and IL-15 by ovarian steroids, TNF-alpha and TGF-beta in human endometrial epithelial and stromal cells Mol Hum Reprod 2005 11 10 751 760 10.1093/molehr/gah233 16254005

