# Supplementary material for: Screening of Variants in the Transcript Profile of Eutopic Endometrium from Infertile Women with Endometriosis during the Implantation Window
Source: Rev Bras Ginecol Obstet. 2021 Jul 27;43(6):457–66. doi: 10.1055/s-0041-1730287 (PMC10411168; doi:10.1055/s-0041-1730287)
Supplement: Supplementary file 1 — Supplementary Material [file 10-1055-s-0041-1730287-s200299.pdf]

Supplemental Table S1 Age, weight, height and body mass index (BMI) of infertile patients with endometriosis, infertile controls and fertile controls

Age (years)	33,7 (29,9–38,9)	35,9 (31,2–40,2)	32,9 (27,9–36,5)	0,52
Weight (Kg)	60,9 (56,0–73,3)	61,6 (53,4–70,8)	74,8 (60,8–82,3)	0,30
Height (m)	1,62 (1,56–1,69)	1,61 (1,57–1,64)	1,62 (1,56–1,72)	0,89
BMI (Kg/m^2)	22,9 (21,6–27,0)	23,8 (20,4–27,7)	27,2 (24,4–29,26)	0,17

Note: data expressed as median (interquartile deviation). Kruskal-Wallis test. Level of significance: 5%.

 © 2021. Federação Brasileira de Ginecologia e Obstetrícia. All rights reserved.
